# Bidirectional Coupler Study for Chip-Based Spectral-Domain Optical Coherence Tomography

**DOI:** 10.3390/mi13030373

**Published:** 2022-02-26

**Authors:** Hong-Yan Zheng, Bo-Liang Chen, Hsiao-Yen Lu, Shih-Hsiang Hsu, Masanori Takabayashi

**Affiliations:** 1 Department of Electronic Engineering, National Taiwan University of Science and Technology, No. 43, Sec. 4, Keelung Rd., Taipei 10607, Taiwan; m10902305@mail.ntust.edu.tw (H.-Y.Z.); m10902316@mail.ntust.edu.tw (B.-L.C.); m10802328@mail.ntust.edu.tw (H.-Y.L.); 2 Department of Physics and Information Technology, Kyushu Institute of Technology, 680-4 Kawazu, Iizuka 820-8502, Japan; 3 Research Center for Neuromorphic AI Hardware, Kyushu Institute of Technology, 2-4 Hibikino, Wakamatsu-ku, Kitakyushu 808-0196, Japan

**Keywords:** waveguide, optical coherence tomography, coupler

## Abstract

A chip-based spectral-domain optical coherence tomography (SD-OCT) system consists of a broadband source, interferometer, and spectrometer. The optical power divider flatness in the interferometer’s wavelength is crucial to higher signal-to-noise ratios. A Mach–Zehnder directional coupler (MZDC) structure could be utilized to smoothly maximize the splitting ratio of 50:50 on a silicon platform, with a sub-micrometer of decoupler optical path difference insensitive to the process variation up to 20 nanometers. However, the optical signal reflected from the reference and sample will go back to the same interferometer MZDC. The so-called bidirectional coupler MZDC will not illustrate a flat optical power response in the operating wavelength range but could still demonstrate at least 20 dB signal-to-noise ratio improvement in OCT after the echelle grating spectrum compensation is applied. For maintaining the axial resolution and sensitivity, the echelle grating is also insensitive to process shifts such as MZDC and could be further utilized to compensate a 3 dB bidirectional MZDC structure for a broad and flat 100 nm wavelength response in the interferometer-based on-chip SD-OCT.

## 1. Introduction

Optical coherence tomography (OCT) is an imaging technique with a resolution in the µm range and depth in the millimeter range. Spectral domain-OCT (SD-OCT) is based on the principle of low coherence interferometry, while swept source-OCT (SS-OCT) uses a wavelength-swept laser. Both OCT technologies need further intensive steps in signal processing. The image resolution is one of the most critical parameters governing OCT image quality. In contrast to standard microscopy, OCT can achieve better axial resolution independent of the beam focusing and spot size. The bandwidth of the light source determines the image resolution [1,2,3,4,5].

In recent years, silicon-on-insulator (SOI) substrates have been utilized for biosensor research. Due to silicon wires’ high refractive index contrast, its photonic device footprint can be significantly reduced. Moreover, the silicon photonic process is compatible with a complementary metal-oxide-semiconductor fabrication, which will benefit the development of high-density optoelectronic integrated circuits.

SOI has been developed as a common substrate for optical and electronic applications, a critical platform for optoelectronic circuits. This platform can transform a bulky OCT system into a compact silicon-photonics chip [6]. We reported the influence of optical power splitting on the sensitivity and axial resolution in SD-OCT on silicon chip using a tandem Mach–Zehnder directional coupler-based broadband coupler through particle swarm optimization (PSO) [7]. However, the PSO optimization is sensitive to the process variation. Here, the process insensitivity approach will be proposed to maintain the sensitivity of SD-OCT on-chip.

**Figure 1:**
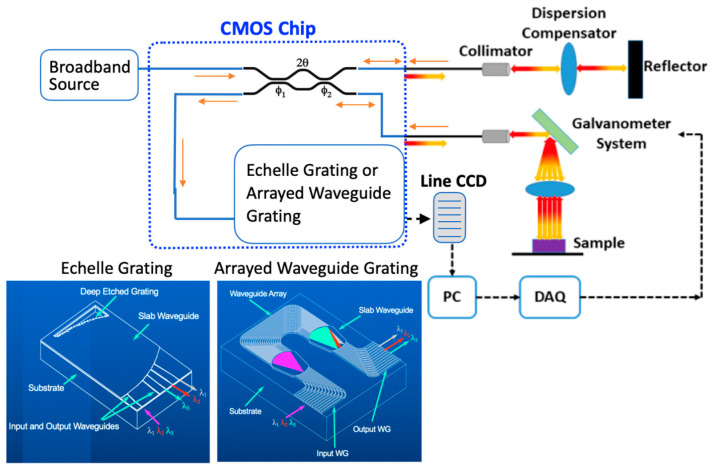
Optical coupler-based SD-OCT interferometer.

A chip-scale SD-OCT system consists of a broad bandwidth light source, interferometer, and spectrometer. In the beginning, the broadband source light is sent to one of the interferometer inputs. Then, its two outputs will be connected to the sample and reflector sides and immediately followed by their reflections treated as the Michelson interferometer inputs. The spectrometer is utilized to filter the recombined signal and inject it into the line charged-coupled device (CCD) array for the interferometric signal analysis through a data acquisition card (DAQ), as shown in Figure 1. In the setup, the collimator and dispersion compensators are two devices utilized for parallel beam coupling and free chromatic dispersion, respectively. They will not be integrated with the silicon chip. Finally, the OCT scanning will go through the Glavanometer system for the structure profiles. In this paper, the application of SD-OCT for the integration of optical components is explored utilizing SOI technology.

One of the crucial components in on-chip OCT is the optical power divider, an essential function, as an interferometer to combine the reflection power from the reference and sample. The most common approach of producing optical power splitting is using a traditional directional coupler (DC) because the proper coupling length adjustment can achieve any arbitrary ratio. This simple design is sensitive to the operating wavelength. According to the coupled-mode theory, the coupling coefficient and propagation constant contribution to the coupling power’s amplitude and phase terms, implying that the coupling ratio performance would vary with the wavelength variation. In the Fourier-domain OCT system, broadband and wavelength-insensitive couplers were proposed and maintained the axial resolution with high signal-to-noise ratios.

In the OCT system, we need a wavelength-insensitive coupler since the Fourier-domain OCT high-axial resolution depends on the overall wavelength response of guided wavelengths. There were several approaches to demonstrate the broadband wavelength response through the optical coupler, such as curved/bent directional coupler [8,9], multimode interference [10], asymmetry [11], adiabatic control [12], bending [13], and genetic process [14]. Compensation manipulation’s decoupled phases developed the process insensitivity with the broadband couplers [15]. Moreover, the high extinction ratio and fabrication tolerance were shown as the dispersion-engineered Mach–Zehnder interferometers [16] and the variable splitter compensation [17]. One of the solutions to obtain a compact, lower insertion loss and flat wavelength-dependent coupler, through the propagation constant and coupling coefficient-related waveguide structures manipulated for the broadband wavelength response, is using a Mach–Zehnder configuration over a directional coupler [18].

Further study shows that the waveguide width and length variation significantly affect the power splitting ratio and wavelength flatness. Moreover, the OCT coupler executes the Michelson interferometer and demonstrates the bidirectional function, in which optical power splitters are intentionally breaking wavelength flatness built from unidirectional parameters. This paper proposes a Mach–Zehnder directional coupler (MZDC) configuration [19] by connecting two DCs through a short delay length for broadband wavelength response. The most common process variation, ±10 nm, will be applied to the MZDC for the effects in OCT. Unfortunately, its bidirectional function in the wavelength is not flat and can be compensated by another process-shift insensitive echelle grating for axial resolution improvement. 

## 2. Theory and Design

**Figure 2:**
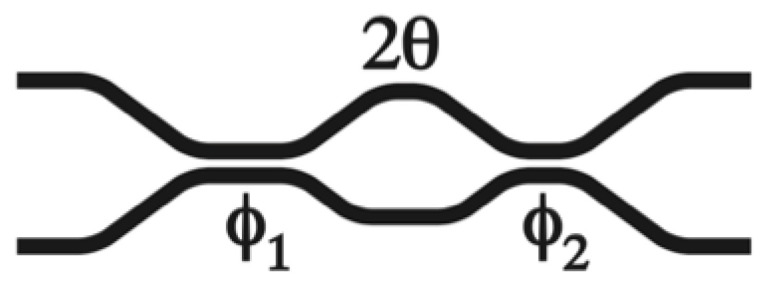
The schematic drawing for the MZDC structure.

A cascade of two similar DCs in a Mach–Zehnder (MZ) configuration [18] was successfully utilized to achieve wavelength-insensitive performance, shown in Figure 2. In addition, its function can be illustrated in the following:

(1) $$\mathrm{MZDC}=DC_{1}*Delay*DC_{2}$$

The matrices of $DC_{1}$ and $DC_{2}$ are represented as follows:

(2) $$DC_{i}=\left[\begin{array}{cc} cos\phi_{i} & -isin\phi_{i}\\ -isin\phi_{i} & cos\phi_{i} \end{array}\right],\;i=1,\;2$$

*Delay* can be in the following:

(3) $$Delay=\left[\begin{array}{cc} 1 & 0\\ 0 & e^{-2i\theta } \end{array}\right]$$

where $\phi_{i}$ and *θ* are for the coupling of *DC_i_* and decoupling phases, respectively. 

The coupling length of the directional coupler, $L_{c}$, can be listed below:

(4) $$L_{c}=\frac{\lambda }{2\left(n_{s}-n_{a}\right)}$$

where *n_s_* and *n_a_* are the effective indices of the two symmetrical and anti-symmetrical supermodes in the coupled straight regions.

(5) $$\theta =\frac{\beta \left(\lambda \right)\Delta L}{2}$$

(6) $$\phi_{i}=\int_{0}^{L_{i}}\frac{\pi }{2L_{c}}dz$$

where *b* is the propagation constant and *l* is the operating wavelength. ∆*L* and *L_i_* represent the optical phase difference in the decoupling and coupling length, respectively. 

**Figure 3:**
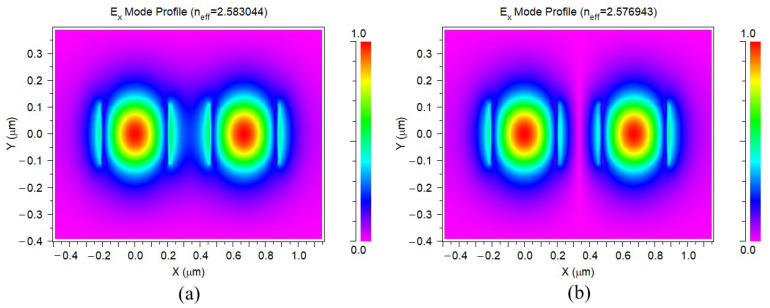
The mode profiles for the (**a**) *n_s_* and (**b**) *n_a_*, under 380 nm width and 220 nm height.

The siliconwire mode profiles for *n_s_* and *n_a_* are simulated under the conditions of 380 nm and 220 nm for width and height, respectively, in the 1310 nm wavelength, shown in Figure 3. We can see the effective index difference is 0.006101, and the coupling length can be estimated as 107 μm. 

The delay length in the MZDC decoupled arms is optimized to obtain the flat wavelength response while maintaining the dedicated splitting ratio. For the MZDC configuration effects in OCT performance improvement, the coupler spectrum is multiplied with Gaussian windows and a single frequency test signal to observe the point spread function (PSF) response, a signal-reflection OCT response. Results indicate that a MZDC with a broad bandwidth response increases the signal-to-noise ratio (SNR) in OCT.

In Figure 1, only one input of the broadband coupler MZDC directs the optical source into two outputs for the sample and reference signals, so two reflections will form the SD-OCT-based interference after the wavelength splitters. Therefore, the MZDC transmission is unidirectional and the reflection is bidirectional. 

The unidirectional MZDC is one input and two outputs. Its transmission matrix can be described as follows:

(7) $$\left[\begin{array}{c} E_{1}\\ E_{2} \end{array}\right]=\left[\begin{array}{cc} cos\emptyset_{2} & -isin\emptyset_{2}\\ -isin\emptyset_{2} & cos\emptyset_{2} \end{array}\right]\left[\begin{array}{cc} 1 & 0\\ 0 & e^{-2i\theta } \end{array}\right]\left[\begin{array}{cc} cos\emptyset_{1} & -isin\emptyset_{1}\\ -isin\emptyset_{1} & cos\emptyset_{1} \end{array}\right]\left[\begin{array}{c} 1\\ 0 \end{array}\right]$$

where $\emptyset_{i}\;\left(i=1,2\right)$ and *θ* are the phases for two directional couplers and one decoupling, respectively.

The unidirectional MZDC outputs, *E*_1_ and *E*_2_, are listed in the following:

(8) $${\left|E_{1}\right|}^{2}=cos^{2}\theta cos^{2}\left(\emptyset_{1}+\emptyset_{2}\right)+sin^{2}\theta cos^{2}\left(\emptyset_{1}-\emptyset_{2}\right)$$

(9) $${\left|E_{2}\right|}^{2}=cos^{2}\theta sin^{2}\left(\emptyset_{1}+\emptyset_{2}\right)+sin^{2}\theta sin^{2}\left(\emptyset_{1}-\emptyset_{2}\right)$$

Two reflective signals, $E_{1}^{\prime }$ and $E_{2}^{\prime }$, will go through the same MZDC from the opposite side and the bidirectional MZDC outputs, *E*_3_ and *E*_4_, can be described as follows:

(10) $$\left[\begin{array}{c} E_{3}\\ E_{4} \end{array}\right]=\left[\begin{array}{cc} cos\emptyset_{1} & -isin\emptyset_{1}\\ -isin\emptyset_{1} & cos\emptyset_{1} \end{array}\right]\left[\begin{array}{cc} 1 & 0\\ 0 & e^{-2i\theta } \end{array}\right]\left[\begin{array}{cc} cos\emptyset_{2} & -isin\emptyset_{2}\\ -isin\emptyset_{2} & cos\emptyset_{2} \end{array}\right]\left[\begin{array}{c} E_{1}^{\prime }\\ E_{2}^{\prime } \end{array}\right]$$

A power splitting ratio of $\varepsilon_{i}$ can be assumed as $sin^{2}\emptyset_{i}$, i being equal to 1 and 2. $E_{3}$ and $E_{4}$ can be derived in the following:

(11) $$\begin{array}{cc} {\left|E_{3}\right|}^{2}=[\left(1-\varepsilon_{2}\right) & \left(1-\varepsilon_{1}\right)+\varepsilon_{2}\varepsilon_{1}-2\sqrt{\left(1-\varepsilon_{2}\right)\varepsilon_{2}\left(1-\varepsilon_{1}\right)\varepsilon_{1}}\cos\left(2\theta \right)]E_{1}^{\prime }{}^{2}\\ & +\left[\varepsilon_{2}\left(1-\varepsilon_{1}\right)+\left(1-\varepsilon_{2}\right)\varepsilon_{1}+2\sqrt{\left(1-\varepsilon_{2}\right)\varepsilon_{2}\left(1-\varepsilon_{1}\right)\varepsilon_{1}}\cos\left(2\theta \right)\right]E_{2}^{\prime }{}^{2}\\ & -2\sqrt{\left(1-\varepsilon_{1}\right)\varepsilon_{1}}\sin\left(2\theta \right)E_{1}^{\prime }E_{2}^{\prime } \end{array}$$

(12) $$\begin{array}{cc} {\left|E_{4}\right|}^{2}=[\left(1-\varepsilon_{2}\right) & \varepsilon_{1}+\varepsilon_{2}\left(1-\varepsilon_{1}\right)+2\sqrt{\left(1-\varepsilon_{2}\right)\varepsilon_{2}\left(1-\varepsilon_{1}\right)\varepsilon_{1}}\cos\left(2\theta \right)]E_{1}^{\prime }{}^{2}\\ & +\left[\left(1-\varepsilon_{2}\right)\left(1-\varepsilon_{1}\right)+\varepsilon_{2}\varepsilon_{1}-2\sqrt{\left(1-\varepsilon_{2}\right)\varepsilon_{2}\left(1-\varepsilon_{1}\right)\varepsilon_{1}}\cos\left(2\theta \right)\right]E_{2}^{\prime }{}^{2}\\ & +2\sqrt{\left(1-\varepsilon_{1}\right)\varepsilon_{1}}\sin\left(2\theta \right)E_{1}^{\prime }E_{2}^{\prime } \end{array}$$

When there are two inputs, the coefficients of the $E_{1}^{\prime }{}^{2}$ and $E_{2}^{\prime }{}^{2}$ are reciprocal from Equations (11) and (12), which can be applied to the term of $E_{1}^{\prime }E_{2}^{\prime }$ only when the splitting functions for two couplers are the same. The reciprocal process for the bidirectional MZDC-based SD-OCT does not exist since the MZDC transmission is one input for the optical source.

**Figure 4:**
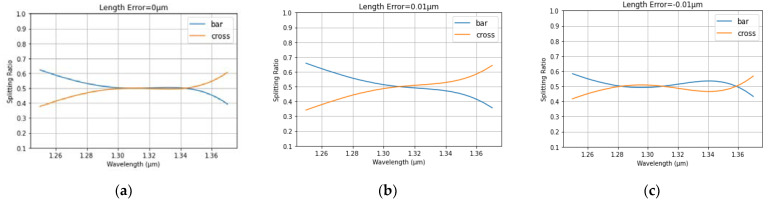
The simulated spectrum response for the unidirectional MZDC (**a**) without the process length shift; (**b**) 10 nm length shift; (**c**) −10 nm length variation.

**Figure 5:**
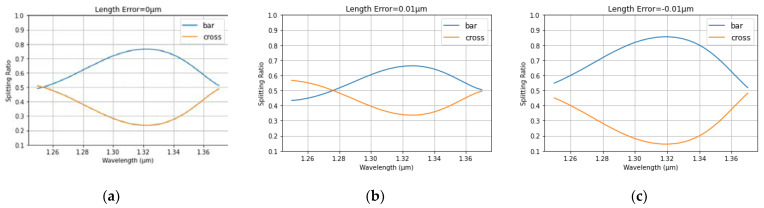
The simulated spectrum response for the bidirectional MZDC (**a**) without the process length shift; (**b**) 10 nm length shift; (**c**) −10 nm length variation.

The spectrum from MZDC is demonstrated considering process variations in the length within ±10 nm, as shown in Figure 2. In addition, the MZDC design parameters are listed in Table 1. A mean percentage error (MPE) of the MZDC coupler performance is calculated by averaging the percentage deviation from the actual optical power response to desired response in each wavelength. The MZDC spectral response in uni- and bi-directional functions is simulated under three process variations in the waveguide length and shown in Figure 4 and Figure 5, respectively. A cross state for MZDC is for the optical power to be completely transferred from the input channel to the other channel at the output. Moreover, the bar state of a MZDC uses the optical strength to completely pass through the input channel at the output without any transfer to the other port.

**Figure 6:**
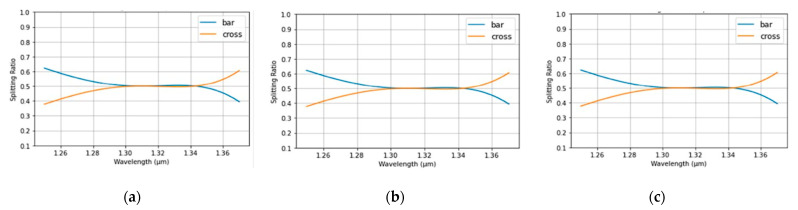
The simulated spectrum response for the unidirectional MZDC is demonstrated in the following situations: (**a**) without the waveguide width variation; (**b**) 10 nm width shift; (**c**) −10 nm width shift.

**Figure 7:**
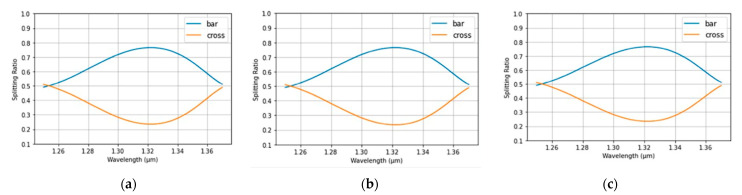
The simulated spectrum response for the bidirectional MZDC is demonstrated in the following situations: (**a**) without the waveguide width variation; (**b**) 10 nm width shift; (**c**) −10 nm width shift.

The waveguide width-error tolerance is considerably ± 10 nm, which are 0.38 μm, 0.39 μm, and 0.37 μm. When the width is varied, the *D*_0_ [1], the phenomenological constant with the μm as the unit, is the only parameter affected in MZDC, and they are 10.92, 11.06, and 10.76 for the widths of 0.38 μm, 0.39 μm, and 0.37 μm, respectively. The spectral response for the unidirectional MZDC is simulated for three process variations in the waveguide width, as shown in Figure 6. In addition, the same simulation is also applied to the bidirectional MZDC, and their spectral are demonstrated in Figure 7. In summary, the length variation is more significant than the width in the MZDC spectrum.

When the signal from the sample and reflector ports is reflected, the splitting ratio performance could be formed an interrogated spectrum from Equation (12). The *E*_4_ field will be connected to the spectrometer, for dealing with the optical power in every wavelength for the SD-OCT data analysis.

The arrayed waveguide grating (AWG) and echelle grating (EG) are two main integrated spectrometers on a chip. For a curved grating of radius R, the diffraction images of a point source located on a circle of radius R/2, tangent to the grating at one point, will be focused on the same circle. This circle is commonly referred to as the Rowland circle. The grating equation for AWG is listed in the following:

(13) $$nd_{AWG}\sin\left(\theta_{i}\right)+nd_{AWG}\sin\left(\theta_{o}\right)+n_{c}\Delta L=m\lambda$$

where *n* and $n_{c}$ are the effective index of the planar and channel waveguides, respectively. Δ*L* is the optical length difference between adjacent channels of the waveguide array. $d_{AWG}$ is the grating period of AWG. $\theta_{i}$ and $\theta_{o}$ are the input and output angles of the diffraction grating. m is the grating order, and λ is the operating wavelength. 

The EG grating equation is AWG, except for the optical phase difference. In addition, it can be represented as follows:

(14) $$nd_{EG}\sin\left(\theta_{i}\right)+nd_{EG}\sin\left(\theta_{o}\right)=m\lambda$$

where $d_{EG}$ is the grating period of EG.

**Figure 8:**
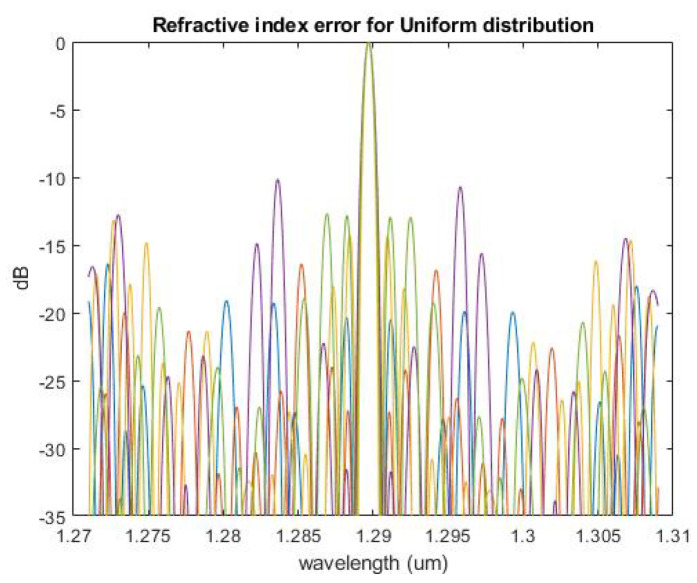
AWG transmission spectrum through the uniform distribution in the waveguide width variation of ±10 nm. Different color lines represent each random number in the waveguide width variation range.

Since the siliconwire experiences the process variation in the waveguide width and length in a ±10 nm range, the waveguide array phase will not be constant and will cause higher crosstalk [20,21]. The AWG transmission spectrum was utilized for crosstalk simulation through the uniform distribution in the waveguide width variation of ±10 nm. The non-adjacent crosstalk is increased up to −10 dBm, as shown in Figure 8. The different colors represent one uniform distribution in the process variation.

## 3. Results and Discussions

**Figure 9:**
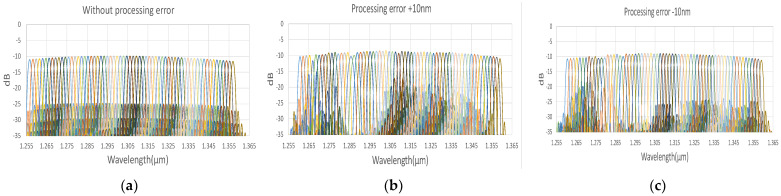
The simulated spectrum response for the AWG (**a**) without the process length shift; (**b**) at 10 nm waveguide width shift; (**c**) at −10 nm waveguide width variation. Different color lines represent individual wavelength output channels.

**Figure 10:**
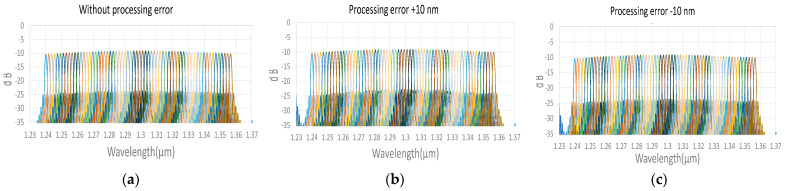
The simulated spectrum response for the EG (**a**) without the process length shift; (**b**) at 10 nm waveguide width shift; (**c**) at −10 nm waveguide width variation. Different color lines represent individual wavelength output channels.

**Figure 11:**
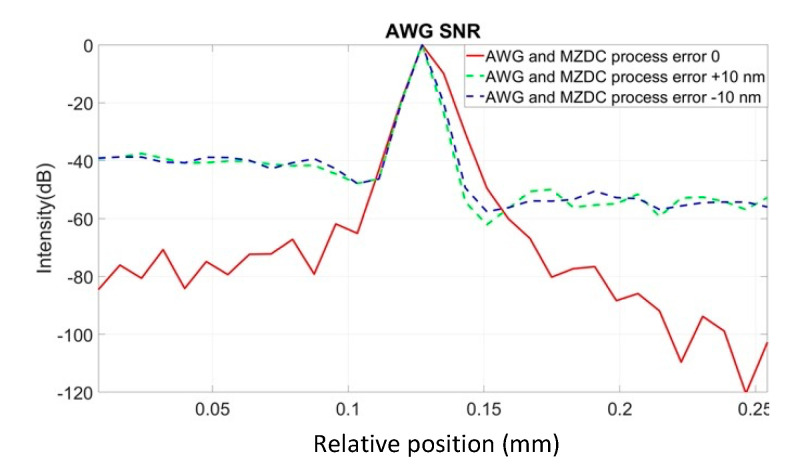
PSF from the MZDC interrogated with AWG.

**Figure 12:**
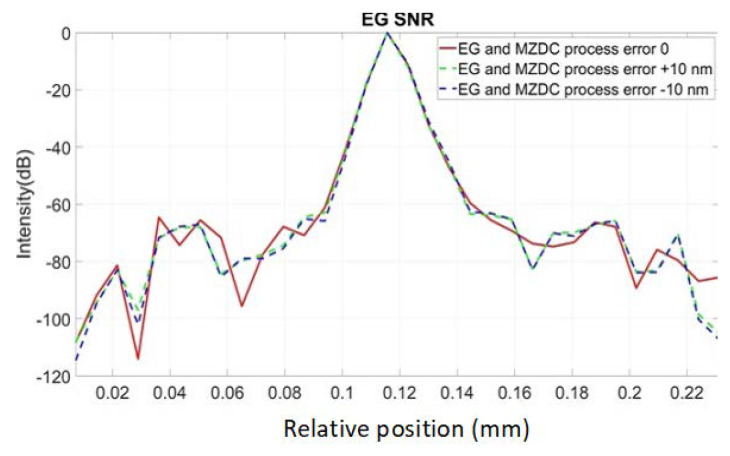
PSF from the MZDC interrogated with EG.

To demonstrate all the channel output spectrum in AWG and EG [22,23,24,25], we used the commercial software of EPIPPROP from Photon Design to simulate the process variation effects in two waveguide length shifts, +10 and −10 nm, shown in Figure 9 and Figure 10. The design parameters for 64-channel AWG and EG in the 1310 nm wavelength range are listed in Table 2. In addition, the phase error from the waveguide array and grating tapers will cause higher crosstalk up to −15 dB in AWG. On the other hand, the EG crosstalk can always be maintained on the same level, −25 dB, due to the phase-error-free in the reflective grating. The EG far-field intensity in Gaussian distribution can compensate for the bidirectional MZDC spectrum to reach better OCT axial resolution through the PSF execution on the interrogated wavelength range. The uneven AWG spectrum under ±10 nm process errors causes the 3 dB linewidth to get narrow, shown in Figure 11. The final flat wavelength response from compensation illustrates almost the exact SD-OCT axial resolution at the full width at half maximum (FWHM) from AWG and EG, shown in Figure 11 and Figure 12. However, the MZDC interrogated EG demonstrates the lowest crosstalk, −80 dBm, as shown in Figure 12, even though the process variation is applied.

**Figure 13:**
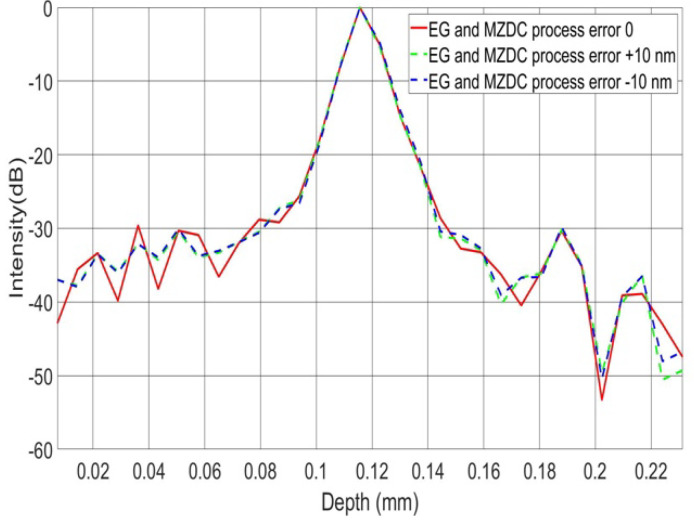
The point spread function only from the bidirectional MZDC with ED compensation.

The EG is used as a spectrometer and a spectral equalizer to compensate for responses of bidirectional MZDC through the non-uniform EG spectrum. When only the bidirectional MZDC effect in the SD-OCT system is considered, the free spectral range of EG can be designed at least two times larger than 120 nm, and its spectrum can be treated as the flat response. Compared with the EG spectral-equalizer function, the PSF only from the bidirectional MZDC demonstrates the higher crosstalk up to 40 dB, shown in Figure 13.

**Figure 14:**
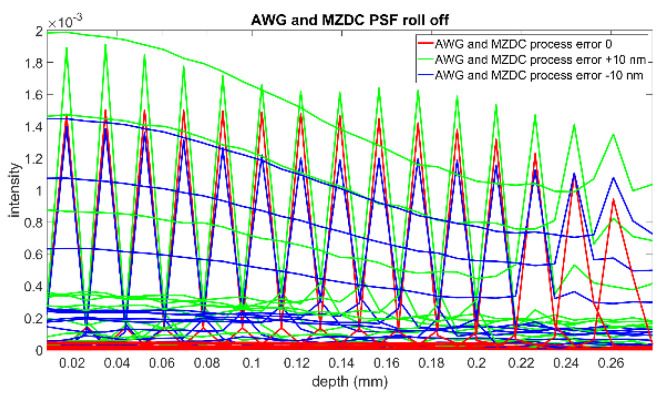
PSF roll-off from the MZDC interrogated with AWG.

**Figure 15:**
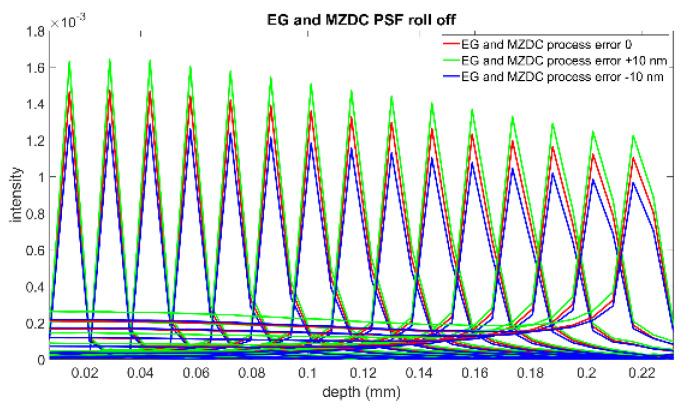
PSF roll-off from the MZDC interrogated with EG.

This interference spectrum distribution in space from the signal and reference signals is called a PSF. The complex-valued depth-dependent OCT signal is obtained from an inverse Fourier transform of the interference [2]. Therefore, the inverse Fourier transform of the source spectrum, the axial PSF convoluted with the sample reflectivity, gives the OCT roll-off amplitude signal. The PSF roll-off simulations for MZDC interrogated with the AWG and EG are shown in Figure 14 and Figure 15, respectively. The results show that the error caused by the AWG severely distorts the PSF at the deeper measurement depths, and the SNR performance is inferior. The EG is less sensitive to ± 10 nm process error, and simulation results demonstrate that the PSF roll-off can maintain superior signal strength and resolution in the maximum measurement range. The SNR of EG is much better than that of the AWG. For retaining the axial resolution and sensitivity, the EG is also insensitive to process shifts such as MZDC and could be further utilized to compensate a 3 dB bidirectional MZDC structure for a broad and flat 100 nm wavelength response on the interferometer-based on-chip SD-OCT.

## 4. Conclusions

To make the free-space SD-OCT system highly integrated, we replaced the fiber-type coupler and diffraction grating by MZDC and EG or AWG on a silicon chip, so that the bar and cross side of MZDC can be output to the sample and reference arms. Then, the coupler-based SD-OCT system executes the Michelson interferometer and accepts the reflected beams from the same sides. At the same time, we found that the spectrum of the reflected beam entering the MZDC from the bar and cross ends is not flat again, so we compensated for this via the 64-channel EG spectrum response, which is insensitive to the process variation. After the PSF simulation, we can observe that the SNR of MZDC combined with AWG is as high as 61.3 dB without process error. Still, after considering the process error, the SNR performance drops significantly to about 39.3 dB. The SNR simulation result of MZDC interrogated with EG shows that the SNR performance can be maintained at a great 64.5 dB with or without process error, so the SNR of MZDC combined with EG is less sensitive to the process errors.

## Figures and Tables

**Table 1:** MZDC design parameters.

Splitting Ratio	50:50
$L_{1}$	112.39 μm
$L_{2}$	54.77 μm
ΔL	0.17 μm
MPE (Mean Percentage Error)	2.99%

**Table 2:** AWG & EG design parameters.

Parameters	AWG	EG
Channel Spacing	1.58 nm	1.85 nm
Input Waveguide Spacing	1.9 mm	5 mm
Rowland Circle	528 mm	1300 mm
FSR (Free Spectral Range)	120 nm	120 nm
m (Grating Order)	6	8
$\theta_{i}$ (Input Angle)	0°	110°
*n* (Effective Index of Planar Waveguides)	2.988	2.988
*n_c_* (Effective Index of Channel Waveguides)	2.516	2.516
D*L* (Optical Length Difference)	3.124 mm	NA
*d_AWG_*	1.51 mm	NA
*d_EG_*	NA	5 mm

## Data Availability

Not applicable.
